# Perspectives on and Quality of Life in Skin of Color Patients With Mycosis Fungoides/Sézary Syndrome: A Qualitative Analysis

**DOI:** 10.7759/cureus.34054

**Published:** 2023-01-22

**Authors:** Jaclyn Abraham, Grace Wei, Seemal R Desai, Pei-Ling Chen, Lucia Seminario-Vidal

**Affiliations:** 1 Department of Medicine, Morsani College of Medicine, University of South Florida, Tampa, USA; 2 Department of Dermatology, University of Texas Southwestern Medical Center, Dallas, USA; 3 Department of Cutaneous Oncology, Moffitt Cancer Center, Tampa, USA; 4 Department of Dermatology and Cutaneous Surgery, Morsani College of Medicine, University of South Florida, Tampa, USA

**Keywords:** skin of color, health disparities, quality of life, cutaneous, t-cells, lymphoma

## Abstract

Background

Prior quantitative studies have described the diminished health-related quality of life (HRQoL) faced by the overall mycosis fungoides (MF)/Sézary syndrome (SS) population; yet, little is known about how the disease affects HRQoL in skin of color (SOC) patients. This qualitative study sought to explore the lived experiences of SOC patients with MF/SS and gain deeper insights into the impact the disease has on various facets of HRQoL.

Methodology

Interviews with SOC patients with MF/SS ≥18 were recruited from a cutaneous lymphoma clinic. A thematic analysis was performed to identify overarching themes.

Results

Ten patients were invited to participate from July to September 2021. One patient with SS and seven patients with MF (four in the early stage and four in the advanced stage), with a median age of 60.5 years, agreed to participate. Emerging themes included diagnostic and therapeutic delays frequently due to initial misdiagnoses with other skin conditions. Physical and functional burdens significantly hindered participants’ abilities to carry out daily responsibilities and maintain employment, and impacts on physical appearance (e.g., darkened skin) led to increased self-consciousness and lack of social acceptance. Participants regarded family and faith as main sources of support in addition to developing healthy coping strategies, such as self-acceptance and adaptability. All participants reported feeling satisfied with their access to healthcare information and the quality of care received.

Conclusions

Our findings provide greater insights into how HRQoL is impacted across SOC patients with MF/SS, which can help raise awareness among healthcare providers and assist with creating interdisciplinary healthcare approaches to better support the needs of this population.

## Introduction

Dermatologic health disparities are most prominent in ethnic minorities and skin of color (SOC) patients [1]. There is increased morbidity and mortality among a range of dermatologic conditions, including cutaneous malignancies [1,2]. For instance, although the incidence of melanoma is highest in non-SOC (NSOC) individuals, SOC patients are more likely to be diagnosed at more advanced stages of the disease and have a significantly reduced five-year survival rate compared to NSOC patients (74.1% versus 92.9%) [1]. These outcome disparities are also observed in patients with nonmelanoma skin cancer, such as squamous cell carcinoma, who commonly have more aggressive tumors and receive a diagnosis further along in the disease process [1]. Mycosis fungoides (MF) and Sézary syndrome (SS) are among the most common subtypes of cutaneous T-cell lymphoma (CTCL), a rare type of non-Hodgkin’s lymphoma characterized by uncontrolled T-cell proliferation predominantly affecting the skin [1,3]. CTCL comprises 2% of all lymphomas, with an approximate incidence rate of 0.3 to 1.0 per 100,000 lymphoma cases annually [4]. Studies have demonstrated that the incidence of MF/SS in the United States is highest in populations with SOC, with prominent differences in disease presentation and outcomes between NSOC and SOC patients with MF/SS, including younger age at clinical presentation and higher mortality rates among SOC patients [1,2,5].

Previous studies have described the negative impacts that CTCL has on patients’ health-related quality of life (HRQoL), which encompasses physical, psychological, and social aspects of health [6-9]. Recently, our group did a survey study that compared HRQoL between SOC and NSOC patients with CTCL and found that the degree of pruritus, skin-related embarrassment, and interference with participation in sports, social, and leisure activities were significantly more pronounced in SOC patients [8]. Due to the lack of qualitative HRQoL research in SOC patients with MF/SS and the disease outcome disparities observed in this demographic, we sought to address this gap by conducting semi-structured interviews and thematic analyses to gather in-depth opinions and identify new patient perspectives that may have been overlooked in prior studies. This article was previously posted to the Research Square preprint server on April 18, 2022; DOI: 10.21203/rs.3.rs-1458724/v1. This article was also previously presented as a meeting abstract at the 2022 American Academy of Dermatology Annual Meeting on March 26, 2022.

## Materials and methods

This qualitative study is reported per the Standards for Reporting Qualitative Research (SRQR) [10]. Ten adult MF/SS SOC patients were invited to participate in a semi-structured interview. The number of participants was not based on statistical calculation, as this study was designed to gain in-depth perceptions and identify novel themes affecting HRQoL of SOC patients with MF/SS. Comparable sample sizes have been used in similar studies [11,12]. The details of the qualitative approach are outlined later.

Recruitment 

Patients were recruited from a single multidisciplinary cutaneous lymphoma clinic at a National Cancer Institute (NCI)-designated cancer center in Florida via a recruitment flyer and intake coordinator during routine clinical appointments. Recruitment from an outpatient setting was performed to enroll patients during a period of disease stability, excluding hospitalized patients with disease progression or complications that may have influenced study results. Established patients over the age of 18 years who were of SOC (Black, Hispanic, or Asian) and English-speaking and had a diagnosis of MF or SS per NCCN guidelines were eligible to participate. SOC was defined broadly as any race or ethnicity other than non-Hispanic White and Fitzpatrick skin types IV through VI [13]. The skin phototype of each study participant was determined by Dr. Seminario-Vidal.

Study design and analysis

An interview script adapted from Beynon et al. was created and utilized for this study (Appendix) [7]. One-on-one, semi-structured interviews were conducted. Patients attending this multidisciplinary clinic travel from geographically distant sites, and to minimize the risk of exposure to COVID-19, interviews were conducted via phone or Microsoft Teams between July and September 2021. To decrease questioning and interpretation biases, interviewers were not known to nor had any preexisting clinical relationship with the participants. Interviewers were supervised by clinic directors with experience in qualitative research who also oversaw data analysis and interpretation. Informed consent was obtained before beginning each interview. All interviews were audio recorded and transcribed verbatim. To protect participant privacy, all interviews were performed privately between the interviewer and participant in an enclosed setting. Interview audio recordings and transcripts were uploaded to a password-protected folder in a Transport Layer Security (TLS) data-encrypted software application. Interviews were performed until novel codes and themes ceased to emerge and all research team members agreed data saturation was reached.

An inductive thematic analysis was performed on collected data to identify recurrent themes throughout the interviews [14]. Preliminary coding of interview transcripts was performed using a data-driven approach to create an initial codebook. Codes were adapted and modified as needed throughout the analysis and the final codebook was approved by the principal investigator with extensive experience with the MF/SS patient population. Two researchers independently coded each transcript using the codebook. Discrepancies were reviewed until a consensus was reached. The codes generated were then organized into overarching themes. Coding for each interview was compared between the two researchers and inter-rater reliability was calculated to ensure consistency (Cohen's κ = 0.8972). 

## Results

Participants' demographics

A total of 10 patients were contacted for interviews, with eight completed. Two interviews were unable to be completed due to one hospital admission and one personal emergency. Eight (four male and four female) patients with a median age of 60.5 (27-79) years participated in an interview ranging from 16 to 57 minutes in length (Table 1). Five participants (four females and one male) identified as Black, and three (three males) identified as Hispanic. Three participants were classified as Fitzpatrick skin type IV, one participant was skin type V, and four participants were skin type VI. Our sample consisted of one patient with SS and seven patients with MF and was distributed equally regarding disease stage (four early (stages IA-IIA) and four advanced (stages IIB-IVA)).

**Table 1:** Participants' characteristics.

Characteristics	Results (*N*)
Age (years)	
18-29	1
30-49	0
50-69	5
≥70	2
Gender	
Male	4
Female	4
CTCL subtype	
Mycosis fungoides	7
Sézary syndrome	1
Disease stage	
IA	3
IB	1
IIB	2
IIIA	1
IVB	1
Race/Ethnicity	
Black	5
Hispanic	3
Fitzpatrick skin type	
IV	3
V	1
VI	4

Thematic analysis

Our analysis identified eight overarching themes that encompass both the positive and challenging aspects of living with MF/SS and its impact on patient HRQoL. These themes include diagnosis experience, physical consequences, psychosocial consequences, financial implications, questions and concerns surrounding diagnosis, healthcare experience, coping mechanisms, and sources of support. A description of major themes, subthemes, and patient quotes to illustrate our findings are described in the following sections, as well as in Table 2.

**Table 2:** Quotations from patients with mycosis fungoides/Sézary syndrome by theme. P, participant; F, female; M, male

Theme: Diagnosis experience	
Subtheme	*n* (%)
Delayed time to diagnosis	4 (50%)
“I've been treated for different kinds of diseases until I came to [cancer center] in my fourth year, and they did the same routine that all the doctors had been doing, but the studies didn't show up, like, between four to five years in the disease.” (P1, F)	
Reaction to diagnosis	8 (100%)
“I didn't, at that point, understand what it really was. I think the question I asked my dermatologist was, is this something that can kill me? And he answered, ‘most people die with it, not of it’. So that, to me, just kind of took the weight off, and I just went about life as if everything was fine.” (P2, M)	
Theme: Physical consequences	
Subtheme	*n* (%)
Pruritus	8 (100%)
“A lot of itchiness on my body. I was scratching all the time.” (P7, M)	
Pain	3 (38%)
“Sometimes, I get like, pins and needles. You know, used to get it, when it was bad, I used to get it all the time, for some reason. Back of my thighs and across my back. It was horrible.” (P8, M)	
Mobility	5 (63%)
“My wrist and my ankles, my, well, my feet swelled up, so it was hard to walk and things like that.” (P3, F)	
Physical appearance	3 (38%)
“The itch was very tormenting, and the deformity of my face was horrible. And the color of my skin darkened tremendously. I didn't look like the same person anymore. I looked like a beast at the beginning, the beginning was beastly.” (P5, F)	
Sleep quality	2 (25%)
“Before treatment it was... waking up, itching, just overall being uncomfortable.” (P3, F)	
Theme: Psychosocial consequences	
Subtheme	*n* (%)
Sense of lost identity	3 (38%)
“I was distraught because I watched myself just turn to another... another individual that I could not I can't explain it, but I was in disbelief to see that I just sit there and watch myself... just change to another person.” (P1, F)	
Self-consciousness	3 (38%)
“People look. You go to a ballgame, my son's a little league game, I sit in the bleachers with shorts and the person next to me looks at my knee. You know? That looks like a rash. Looks like, you know...” (P8, M)	
Social isolation	3 (38%)
“It affected my relationship with my friends. My coworkers, they, a lot of [them] cut themselves off, you know, from me. They stopped communicating with me. I would call, and they would not answer the phone.” (P5, F)	
Theme: Financial implications	
Subtheme	*n* (%)
Loss of employment	3 (38%)
“I didn't retire. I was let go. Right. And then because, then it came to a thing where I couldn't work these days, because [Doctor's name] had me there three days a week. So, it just turned out that I can't come into work.” (P8, M)	
Healthcare costs	2 (25%)
“Finances... yeah, 'cause doctor bills start rolling in.” (P3, F)	
Theme: Questions and concerns surrounding diagnosis	
Subtheme	*n* (%)
Disease etiology	4 (50%)
“I guess, probably like anyone else, just, what does it stem from? Basically, yeah, what is, what causes it? Yeah, basically, what's, what's the root cause of the disease?” (P4, F)	
Lack of cure	3 (38%)
“Like, the way they will spend money for other cancers, you know, they would give big money for other cancers to be researched, researched more. I don't think, I don't think cutaneous T-cell lymphoma is getting that acknowledge, that acknowledgement from the government. Because, because it's not, it's like, a rare disease, but a lot of people are having it now.” (P1, F)	
Time to diagnosis	2 (25%)
“If it was a perfect world... I would have liked to have been listened to. I felt like one of the reasons my diagnosis took as long as it did, like, to form a diagnosis, is because I'm a black woman in America, and I feel like black women aren't taken serious in the medical field.” (P3, F)	
Theme: Healthcare experience	
Subtheme	*n* (%)
Access to information	7 (88%)
“I feel I can pick up the phone and get an answer to whatever question I might have. So, that's very reassuring.” (P2, M)	
Quality of care	5 (63%)
“… [H]is bedside manner is, he's like, so enthusiastic about treating you, you know? And like I said, detail, it was so, I mean, he went through my whole PET scan. I had a PET scan done from my forehead to just above my knees. And he went through every single inch of that PET scan with me and my wife in full detail.” (P8, M)	
Multiple therapies	3 (38%)
“’You did this one. You did that. Oh, you had an allergic reaction. Okay, now this is the next one’... am I a guinea pig or something? What are we, trying me out?” (P5, F)	
Theme: Coping mechanisms	
Subtheme	*n* (%)
Self-acceptance	3 (38%)
“I had to get to a part where I acknowledge what it was and stop being in denial, just acknowledge and say, okay, it's there. I have to find a way to live with it and don't let it be a part of me.” (P1, F)	
Adaptability	5 (63%)
“No, no, I mean, it, it gets me a little disappointed when I realize I can't do everything I used to do, but other than that, you know, you just try a little harder, that's all.” (P2, M)	
Sharing experience	3 (38%)
“I appreciate you giving me the opportunity to share. And actually, it really felt good, you know, explaining myself in the questions and it just... I felt good about it, about sharing what I've been going through for the last five years.” (P5, F)	
Theme: Sources of support	
Subtheme	*n* (%)
Family	6 (75%)
“My wife and my three sons...” (P7, M)	
Medical professionals	5 (63%)
“I mean, health professionals, definitely, I think I have a great team of people working around me. I couldn't say more about them.” (P2, M)	
Faith	4 (50%)
“I guess that's why I don't really worry, or it doesn't really bother me. Because, you know, I know it's in God's hands. And I mean, what else can I do but do what I need to do?” (P4, F)	
Therapist	1 (13%)
“Having a support system, seeing a therapist, my faith.” (P3, F)	

Diagnosis experience

The diagnosis experience recounted by participants fell into two subthemes: time to diagnosis and reaction to diagnosis. Half of our participants reported a delay in diagnosis, frequently as a consequence of an initial misdiagnosis with another skin disorder: “…[T]hey couldn't pinpoint what was wrong with me for other... dermatitis, psoriasis, lupus. I've been treated for different kinds of diseases until I came to [cancer center] in my fourth year…” (P1, female). Delays typically ranged between two to four years in length and often required participants to visit multiple healthcare providers (“[W]hen I was first diagnosed, the doctor said he didn't believe it. And then it went like another two years, I think, before I really got seen because I switched dermatologists. And that dermatologist started treating me when he realized that maybe it was a little more serious, and he should send me to a specialist in the city.” (P2, male)), whereas the diagnosis for others was fairly straightforward (“He said that this may be a T-cell lymphoma or Mycosis Fungoides. Let's do a biopsy. Boom, biopsy came back.” (P7, male)). One participant emphasized the frustration they felt when learning they had been receiving treatments for the wrong disorder, which contributed to a temporary skepticism towards their healthcare providers: “How did I feel? Angry. I guess I was angry. I was, you know, you start questioning the doctors, you know. Their experience, their expertise. How can they miss it for so long, you know?” (P8, male). 

The reactions of participants when notified of their diagnosis ranged from feelings of relief, indifference, sadness, fear, and mixed emotions. For some who had dealt with years of uncertainty and inadequate treatments due to an initial misdiagnosis, a definitive diagnosis offered relief: “... [I]t was a relief, because now that I found out what it is, then it could, you know, be treated properly, you know?” (P1, female). Other participants felt indifferent when learning of their diagnosis, choosing to focus their energy on learning as much as they could about the disease: “Um, I guess I really didn't feel one way or another. I didn't feel bad. I didn't feel happy. I just kind of felt normal. I mean, I guess I kind of prepared myself for whatever the diagnosis would be.” (P4, female). Sadness (“[I] was a little bit sad.” (P6, male)) and fear (“They tell you, ‘You have cancer.’ You first panic.” (P8, male)) were other reactions felt by participants, but a clear explanation of the disease, prognosis, and treatment plan by the provider usually dissipated any uneasy feelings.

Physical consequences

Pruritus was a physical symptom experienced by all participants, ranging in severity from mild to severe (“The itch was very tormenting.” (P5, female)) and interfering with some participants’ sleep (“Before treatment it was... waking up, itching, just overall being uncomfortable.” (P3, female)). Some participants noted pain as a result of their skin condition, including neuropathic symptoms such as “pins and needles” (P8, male). The majority of participants reported that their mobility was impacted to some degree: “…my feet swelled up, so it was hard to walk and things like that.” (P3, female). Other physical effects of the disease involved participants’ physical appearance, including skin discoloration and substantial swelling. 

Psychosocial consequences

Because MF/SS manifests on the skin, many participants expressed feelings of self-consciousness, isolation, decreased social activity, lack of social acceptance, loss of identity, and general feelings of unattractiveness. For example, the isolation described by some participants usually resulted from self-consciousness, as described by P1 (female): “It has me stay in my house and [not] want to go outside because... when I stare in the mirror, I know, I see, like, a walking dead, I'm telling you.” Some participants also mentioned intentionally wearing long sleeves and pants to hide their condition. Other participants described a loss of identity from the inability to carry out responsibilities, in some instances leading to an increased burden on their families: “You feel bad, as a father, as a husband, as a man, that you're not doing your obligations, you know?” (P8, male). One participant encountered a lack of acceptance in the form of a hostile work environment, which led to leaving work and losing coworkers they had once considered friends: “I dealt with students. They were very cruel. And some of the staff is very cruel, too… like, 'Oh, get away from me, oh, look at you, look at your skin’.” (P5, female). 

Financial implications

Loss of employment and healthcare costs contributed to a financial burden some participants experienced as a result of their disease. Loss of employment resulted from many factors, including physical symptoms (e.g., fatigue and painful sores) and the time commitment required for treatment: “I was let go… because, then it came to a thing where I couldn't work these days, because [doctor's name] had me there three days a week.” (P8, male). Costs associated with healthcare visits (“…doctor bills start rolling in.” (P3, female)) and treatments were also sources of stress. 

Questions and concerns surrounding diagnosis

Many participants mentioned being left with unanswered questions surrounding the etiology of their disease: “Probably, like anyone else, just, what does it stem from? Basically, yeah, what is, what causes it?” (P4, female). Others expressed concerns about the lack of a cure; similarly, trials of multiple treatments required to find an effective treatment also led to frustration (“Am I a guinea pig or something? What are we, trying me out?" (P5, female)). Another concern for participants involved the timing of their diagnosis, with one participant directly attributing their diagnostic delay to SOC discrimination in healthcare: "I felt like one of the reasons my diagnosis took as long as it did… is because I'm a black woman in America, and I feel like black women aren't taken seriously in the medical field.” (P3, female). 

Healthcare experience

All participants reported satisfaction with the current care they were receiving, specifically regarding their access to information (“I feel I could pick up the phone and call somebody and get an answer.” (P2, male)) and the high quality of care provided (“…he went through every single inch of that PET scan with me and my wife in full detail." (P8, male)). Although all participants were pleased with their current care, those who had reached a stage of successful management after multiple years of trial and error highlighted the challenging nature of the experience. For one participant, it took nearly 11 years to find an effective treatment, resulting in questioning the lack of research being put forth into finding a cure for MF/SS: “…the way they will spend money for other cancers, you know, they would give big money for other cancers to be researched… I don't think cutaneous T-cell lymphoma is getting that acknowledgment from the government.” (P1, female). Another participant mentioned frustration with recurrent biopsies needed to monitor their disease, especially on more noticeable areas of the body that led to increased self-consciousness, such as the face (“I have one that was done on my eyebrows, and that, that, that dent is still there on my face, you know…” (P5, female)).

Coping mechanisms

Many participants described coping with the chronicity of their disease through self-acceptance and adaptability (“I would wear, like, long sleeves and always have long pants on. I would not go to the swimming pool, and I would go to the beach, but maybe at night." (P1, female)) and removing focus from events that are out of their control (“What will happen, will happen. I've had a wonderful life. Why should I worry? I'm gonna die no matter what.” (P7, male)). Others found it comforting to share their experiences verbally (“I appreciate you giving me the opportunity to share. And actually, it really felt good, you know, explaining myself in the questions…” (P5, female)) or through written mediums; one participant described a book they are writing about their journey. 

Sources of support

Major sources of support mentioned by participants included family, especially spouses and children (“My wife and my three sons…” (P7, male); “[A]s long as my wife is around, you know…that's the answer to everything.” (P2, male)). Others mentioned medical professionals as an important source of support, particularly due to their ability to provide answers and guidance when necessary (“When I would sit there at every three-month visit, and we would discuss what's going on, I always left there thinking there's a game plan.” (P8, male)). Further, participants also mentioned their religion and faith as a source of comfort (“I guess that's why I don't really worry, or it doesn't really bother me. Because, you know, I know it's in God's hands.” (P4, female)). Two participants mentioned the utilization of a professional therapist or support group, but did not find them to be as effective as their other support systems, such as speaking with family members (“…[Y]ou go into a support group and you're there with them for the day… I am around my family that see me in and out, and they know my good days and my bad days, so we can talk about it, you know?” (P1, female)).

## Discussion

Our qualitative analysis supports prior research evidence that MF/SS is associated with profound impacts on HRQoL and further offers the unique contribution of situating these findings in the context of SOC patients with MF/SS [6,7,9,15-17]. Participants reported significant delays in diagnosis that were often a consequence of initial misdiagnosis, which is consistent with findings from previous CTCL research [2,6]. However, one female participant attributed her delayed diagnosis to healthcare system discrimination against patients with SOC, which represents a novel concern among patients with CTCL. A prior study found that SOC patients with cancer have less confidence in healthcare providers as a result of experiences across their life course (e.g., perceived racial discrimination) and experiences with the healthcare system as a whole (e.g., suspicion of medical care) [18]. Indeed, a study by Gorbatenko-Roth et al. also found that SOC patients receiving dermatologic care in NSOC-designated clinics compared to SOC-designated clinics perceived medical providers to be less thorough [19]. For instance, NSOC clinical providers were perceived as avoiding physical contact during clinical encounters, which could be interpreted as a sign of disrespect and raises concern surrounding provider racial sensitivity [19]. There has also been an association observed between SOC patients with MF and longer, more costly inpatient hospitalizations compared to NSOC patients with MF [20]. This could be the result of disparities in access to ambulatory care or resistance to seeking timely care due to a lack of medical provider trust, leading to greater disease severity requiring inpatient care [20].

The most prominent symptoms described in this study and across the literature included pruritus, pain, impaired ability to perform activities of daily living (ADL), increased self-consciousness, social withdrawal, and a sense of lost identity [6,7,9,17]. Although changes in skin texture and increased erythema have been reported as psychosocial stressors for patients with CTCL in prior studies overall, participants in our SOC cohort additionally reported that the darkening of their skin tone significantly contributed to increased feelings of self-consciousness [6,7,9].

Financial costs associated with cutaneous lymphomas are one of the highest across all dermatologic diseases [21,22]. Some participants indicated that employment loss, frequently due to time commitments required for certain treatments, was a financial burden. Others expressed frustration towards the length of time it took to find effective therapy, citing that the trial-and-error approach was both tiring and financially burdensome. Further, none of the participants were aware of or received any type of financial support.

In the past decade, cancer death rates have declined for common cancers (e.g., lung cancer and breast cancer); however, there is a lack of such success in rare cancers, particularly in minority groups [23,24]. Thus, another source of concern for our participants relates to the unknown cause of the disease and the minimal research efforts, compared to other cancers, to find a cure, which is consistent with other QoL studies in patients with CTCL [6]. In previous qualitative studies that explored QoL among patients with more common types of cancers, participants' concerns surrounding the lack of research efforts toward discovering a cure were reported to a lesser degree [25,26].

Satisfaction with care and access to information were both positive aspects of the illness experience among our participants. Participants reported that ease of access to information and timely communication with members of the healthcare team enhanced their healthcare experience. Similar CTCL QoL studies have also noted that patients who had adequate access to information found that it helped to alleviate their anxieties surrounding their diagnosis [6,7]. This also contributed to participants regarding their healthcare providers as a source of support. Other important support systems mentioned by participants included family, religion, and faith. Some participants reported that sharing their experiences helped them to cope with their disease, while others coped by accepting their circumstances and adapting to the situation. Participants from other CTCL QoL studies have reported that practical support from family and friends would be helpful, such as applying topical medications, driving to doctors’ appointments, and helping with household upkeep [17]. Interestingly, none of our participants reported utilizing resources available from large support groups such as the Cutaneous Lymphoma Foundation or the Lymphoma and Leukemia Society, which raises an important question as to whether this is because they are unaware that such resources exist or because they are uncomfortable with requesting access to these resources.

Our findings highlight the need to increase provider awareness and develop specific resources to address the physical, psychosocial, and financial burdens that impact SOC patients with MF/SS. About the prevalent diagnostic delays among this population, enhancing dermatologists’ training in identifying skin pathologies in non-white skin tones, including expanding the scope of dermatologic learning resources (e.g., textbooks) to include more pictures of pathologies in SOC, could help minimize this pattern of diagnostic delays [27]. Also, utilizing patient-reported outcome measures (PROMs) within the clinical setting may assist with the earlier identification of patients in need of additional support to manage the physical, psychological, and social consequences of their disease [28,29]. Further, increased interdisciplinary clinical management may be beneficial, as previous studies have emphasized the association between psychosocial interventions (e.g., cognitive behavioral therapy, acceptance and commitment therapy, etc.) and improved QoL in patients living with a chronic skin condition [30].

Limitations of our study included small sample size, exclusion of non-English-speaking patients, and possible interviewer bias. Efforts were made to mitigate interviewer bias, including the utilization of a standardized semi-structured interview guide that was employed across all participants. Further, participants were recruited from a tertiary cancer center, which may have a more complex patient population and limit generalizability to all SOC populations with MF/SS. However, we were able to recruit patients with early disease stages, and therefore, the impact on those having local care would be similar. Additional qualitative studies with larger sample sizes drawn from diverse sample populations are needed to better understand the full spectrum of experiences of SOC patients with MF/SS.

## Conclusions

The disease manifestations of MF/SS have significant impacts on the physical, psychological, and social HRQoL of patients. Our findings build upon prior research demonstrating divergent outcomes between NSOC and SOC MF/SS populations and provide a qualitative understanding of the lived experiences of SOC patients with MF/SS. Overarching themes included diagnosis and healthcare experience, physical and psychosocial consequences, financial impacts, and support systems. These findings lend valuable insights into developing interdisciplinary approaches that healthcare professionals can utilize to raise awareness and better support the needs of SOC patients living with CTCL.

## Appendices

**Table 3:** CTCL patient interview guide. This interview guide was extracted and modified from the work of Beynon et al. [7]. CTCL, cutaneous T-cell lymphoma

Topics	Questions
Section 1: General	
Demographics	How old are you (years)?
	How would you describe your ethnicity?
	Gender (Male/Female/Other)
	How would you describe your relationship status? (Married/Cohabiting/Single/Divorced/Widowed)
	How would you describe your employment situation? (Full time/Part time/Retired/Unemployed)
	If employed - What kind of work do you do?
History of Illness	Could you tell me a little bit about your experience when you received your diagnosis? How did you feel?
	Were there any delays, and if yes, could you tell me about that? How long?
	How do you describe the disease to other people if they ask?
Section 2: Living with CTCL	
Physical	Could you tell me about any physical problems you have had as a result of CTCL? Prompts: Pain/Dressing changes/Other symptoms (e.g., itch)
Effect of treatment and any problems	Could you tell me about your experience when receiving treatment? Prompts: Effects on activities of daily living/Psychological impacts/etc.
Lifestyle, social, and relationships	Could you tell me how the disease has affected your life more generally? Prompts: Work/Leisure or social activities/Relationships/Finances/Faith
Communication and information	Have you had any concerns about the way that people have communicated with you? Prompts: Who - health professionals or family/Content or manner of conversations/Accessing people
	Are there any conversations you would have liked to have had that you haven’t been able to? Any conversations you would have preferred not to have? (Why?)
	Have you had any concerns about the amount of information you have been given? Prompts: Amount (too much or little)/Timing (too early or late/too quickly)
Psychological	Has the disease had any impact on how you feel? Or how you feel about yourself? Prompts: Low or anxious/How do you look?
	What changes in your skin have you noticed?
	Has there been any impact on the clothes you can wear?
The future and preferences for care	What have you been told about how your disease is likely to progress? Is there anything about that which makes you particularly worried (or fearful)?
	Is there anything you would particularly like to know about your disease that you have not been told or been too worried to ask?
	Is there anything particular you would like to say about where or how you would wish to be cared for either now or in the future?
	Some people consider making an advanced directive to make it clear what their wishes are. Is this something you would or have considered?
	How satisfied are you with the current care you are receiving? Prompts: Very satisfied/Satisfied/Some concerns/Very dissatisfied
Support	What have you found helpful in supporting you through the disease? Prompts: Medical/Nursing/Support group/Psychologist/Other
	How would you like the services to work differently from the way they do now?
